# Utility of serum IGF-1 for diagnosis of growth hormone deficiency following traumatic brain injury and sport-related concussion

**DOI:** 10.1186/s12902-018-0247-1

**Published:** 2018-04-02

**Authors:** Kirstie Lithgow, Alex Chin, Chantel T. Debert, Gregory A. Kline

**Affiliations:** 1 Division of Endocrinology, Department of Medicine, Cumming School of Medicine, University of Calgary, 1820 Richmond Rd SW, Calgary, AB T2T 5C7 Canada; 2 Clinical Biochemistry, Calgary Laboratory Services and Department of Pathology and Laboratory Medicine, Cumming School of Medicine, University of Calgary, 9, 3535 Research Road NW, Calgary, AB T2L 2K8 Canada; 3 Division of Physical Medicine and Rehabilitation, Department of Clinical Neurosciences Cumming School of Medicine, University of Calgary, 2500 University Dr. NW, Calgary, AB T2N 1N4 Canada

## Abstract

**Background:**

Growth hormone deficiency (GHD) is a potential consequence of traumatic brain injury (TBI), including sport-related concussion (SRC). GH stimulation testing is required for definitive diagnosis; however, this is resource intensive and can be associated with adverse symptoms or risks. Measurement of serum IGF-1 is more practical and accessible, and pituitary tumour patients with hypopituitarism and low serum IGF-1 have been shown to have a high probability of GHD. We aimed to evaluate IGF-1 measurement for diagnosing GHD in our local TBI population.

**Methods:**

We conducted a retrospective chart review of patients evaluated for GHD at the TBI clinic and referred for GH stimulation testing with insulin tolerance test (ITT) or glucagon stimulation test (GST) since December 2013. We obtained demographics, TBI severity, IGF-1, data pertaining to pituitary function, and GH stimulation results. IGF-1 values were used to calculate z-scores per age and gender specific reference ranges. Receiver operator curve analysis was performed to evaluate diagnostic threshold of IGF-1 z-score for determining GHD by GST or ITT.

**Results:**

Sixty four patient charts were reviewed. 48 patients had mild, six had moderate, eight had severe TBI, and two had non-traumatic brain injuries. 47 patients underwent ITT or GST. 27 were confirmed to have GHD (peak hGH < 5 μg/L). IGF-1 level was within the age and gender specific reference range for all patients with confirmed GHD following GH stimulation testing. Only one patient had a baseline IGF-1 level below the age and gender specific reference range; this patient had a normal response to GH stimulation testing. ROC analysis showed IGF-1 z-score AUC f, confirming lack of diagnostic utility.

**Conclusion:**

Baseline IGF-1 is not a useful predictor of GHD in our local TBI population, and therefore has no value as a screening tool. TBI patients undergoing pituitary evaluation will require a dynamic test of GH reserve.

## Background

Growth hormone deficiency (GHD) is an increasingly recognized potential consequence following traumatic brain injury (TBI) [1, 2] Patients with GHD may present with impaired concentration, memory loss, low energy, depression, anxiety, social isolation, and poor quality of life [1, 3]. Due to the subtle and non-specific nature of these symptoms, as well as the potential overlap with neurologic and psychiatric sequelae of TBI, biochemical testing is crucial in making the diagnosis of GHD [2, 4]. GH stimulation testing is required for definitive diagnosis of GHD; the insulin tolerance test (ITT) is considered the reference standard and the glucagon stimulation test (GST) is an acceptable alternative, however, this testing is time consuming, resource intensive, and can be associated with adverse side effects [5]. Measurement of serum IGF-1, as a potential marker of GH activity, is comparatively more practical and accessible, and hypopituitary patients with low serum IGF-1 values have been shown to have a high probability of GHD [6]. Previous authors showed that in a population comprised of patients with moderate and severe TBIs, an IGF-1 greater than 175 ng/mL had high negative predictive value for true GHD according to a stimulation test with peak GH response of < 3 μg/L, and was therefore helpful in deciding which patients should undergo dynamic testing [7]. However, the IGF-1 method changed from a manual to an automated method during this study, and these findings have not been validated in a population that includes patients with mild, moderate, and severe traumatic brain injuries or sports-related concussion (SRC). The latter point is especially relevant considering patients with mild TBI also have significant risk of neuroendocrine dysfunction [8]. Our primary objective was to evaluate the performance of IGF-1 as a screening tool for GHD in a patient population that includes mild (including SRC), moderate, and severe TBI.

## Methods

The procedures followed in this study were in accordance with the ethical standards of the Conjoint Health Research Ethics Board at the University of Calgary, Calgary, Alberta, Canada. We conducted a retrospective review of the electronic medical record of patients referred to endocrinology from the Calgary Brain Injury Program from December 2013–2016 for evaluation of hypopituitarism/GHD with either an ITT or GST. From each patient chart, we collected demographic information including age, sex, weight, and the number of months from injury to endocrinology assessment. We also collected data pertaining to the nature of the TBI including severity (mild, moderate, or severe) and whether the TBI had been classified as a SRC. Severity of injury was classified based on the Mayo Clinic Classification which includes initial GCS, length of loss of consciousness or length of post-traumatic amnesia to retrospectively determine TBI severity [9]. Baseline IGF-1 values were recorded, as well as all biochemical data pertaining to pituitary function. IGF-1 values were used to calculate a z-score for each patient per age and gender specific reference ranges. ITT and GST were performed in a dedicated endocrine testing unit with trained endocrine nurses according previously published standard protocols [10]. The choice of ITT or GST was made according to the availability of sufficient nursing staff as two nurses are required to perform the ITT. A successful insulin hypoglycemic test was determined by the achievement of a nadir blood glucose of < 2.5 mmol/L and all tests achieved this standard. Peak GH response following dynamic GH testing with ITT or GST was used to define the presence or absence of GHD where a peak hGH < 5.0 μg/L was considered abnormal and peak < 3.0 μg/L considered severe GHD. Using the dynamic test as the reference standard, the sensitivity and specificity of IGF-1 levels and IGF-1 Z-score to detect GHD was determined.

### Assays

#### Insulin-like growth factor-1 (IGF-1) method

Serum IGF-1 was measured by a one-step immunometric (sandwich) chemiluminescent immunoassay using the Diasorin Liaison XL platform (DiaSorin, Stillwater, MN, USA). IGF-1 is separated from binding proteins and is captured by a murine monoclonal antibody coated onto solid-phase magnetic particles and a secondary murine monoclonal antibody conjugated with an isoluminol derivative to form an immune complex. After incubation, the unbound material is removed with a wash cycle and starter reagents are added to induce a flash chemiluminescence reaction which is measured by a photomultiplier and is directly proportional to the IGF-1 concentration (ug/L) in the serum. During the period of the study, the assay exhibited imprecision levels of < 8.4%. Age- and gender-specific reference intervals (95th percentile) were adapted according to the manufacturer’s instructions for use. This assay is referenced to the 1st WHO International Standard for Insulin-like Growth Factor-I NIBSC code: 02/254.

#### Growth hormone method

Growth hormone was measured by a one-step immunometric chemiluminescent immunoassay using the Siemens Immulite 2000 XPi platform (Siemens Healthcare Diagnostics, Tarrytown, NY, USA). Growth hormone is captured by a murine monoclonal antibody coated onto the solid phase bead a rabbit polyclonal antibody conjugated with alkaline phosphatase. After incubation, unbound material is removed by a centrifugal wash and chemiluminescent substrate is added to produce a signal which is directly proportional to the growth hormone concentration (ug/L). During the period of the study, the assay exhibited imprecision levels of < 3.9%. Reference intervals (95th percentile) were adapted according to the manufacturer’s instructions for use. This assay is referenced to WHO NIBSC 2nd International Standard 98/574.

### Statistics

Standard descriptive statistics were used to define demographic variables. Group medians were compared using the Mann-Whitney U test. Statistical significance was set at *p* = 0.05. Receiver operator curve analysis was performed to evaluate diagnostic threshold of IGF-1 z-score for determining GHD by GST or ITT. All statistical calculations were performed using SPSS software version 24 [11].

## Results

Sixty four patient charts were retrieved. Baseline demographics according to diagnostic GH status are presented in Table 1. There were 48 mild, 6 moderate, and 8 severe TBIs. Two were excluded from analysis due to having brain injuries of non-traumatic etiology. One patient passed away during the interval between assessment and dynamic testing. 14 patients did not receive GH stimulation testing after endocrinology assessment. Of these patients, six were not felt to have symptoms in keeping with GHD at the time of endocrinology assessment so were not referred on for testing. Five patients declined testing due to improvement in their symptoms at the time of endocrinology assessment, or unwillingness to undergo testing procedures. Three agreed to testing but were lost to follow-up before testing was performed. Of the 47 patients that underwent GH stimulation testing with ITT (*n* = 24, 51%) or GST (*n* = 23, 49%), 27 (57%) were confirmed to have GHD (based on a peak GH level of 5 μg/L or less), 18 (38%) had normal results to stimulation testing. Two patients (4%) patients had borderline results with a peak GH levels of 5.2 and 5.5 μg/L following GST and were classified as GHD at the discretion of the treating clinician, as therapy was offered to these patients due to symptomatology in keeping with growth hormone deficiency. Of the patients with confirmed GHD, 20 (43%) had severe GHD with peak GH < 3.0 μg/L or less.

**Table 1:** Baseline Demographics

	All patients *n* = 62	GH deficient *n* = 29	Non-GH deficient *n* = 18
Age (median, IQR)	43 (21.8)	46 (17)	39 (18) *p* = 0.216
Gender	M: *n* = 29	M: *n* = 15	M: *n* = 7
	F: *n* = 33	F: *n* = 14	F: *n* = 11
Weight (median, IQR)	78.1 kg (32.3)	83 kg (34.0)	74.2 kg (23.6) *p* = 0.220
#months to endo referral (median, IQR)	20 (16)	24 (13)	15 (20) *p* = 0.317
TBI Severity	Mild *n* = 48	Mild *n* = 22	Mild *n* = 17
	Mod *n* = 6	Mod *n* = 1	Mod *n* = 1
	Severe *n* = 8	Severe *n* = 6	
Documented Sport Concussion	*n* = 15	*n* = 7	*n* = 5
	Hockey n = 4	Hockey n = 3	Basketball *n* = 1
	Cycling *n* = 2	Cycling *n* = 2	Dirt biking *n* = 1
	Basketball *n* = 2	Basketball *n* = 1	Football *n* = 1
	Soccer *n* = 2	Football *n* = 1	Equestrian *n* = 1
	Football *n* = 2		Soccer *n* = 1
	Dirt biking *n* = 1		
	Wrestling *n* = 1		
	Equestrian *n* = 1		
Documented pituitary deficits	*n* = 4	*n* = 2	*n* = 1
	*n* = 1 secondary hypothyroidism	*n* = 1 secondary hypothyroidism	*n* = 1 secondary hypogonadism and hypothyroidism
	*n* = 2 secondary hypogonadism	*n* = 1 secondary hypogonadism	
	*n* = 1 secondary hypothyroidism and secondary hypogonadism		
IGF-1 Z score (median, IQR)	−0.193 (2.07)	−0.214 (1.52)	−0.060 (1.24) *p* = 0.979
Peak GH during stim test (median, IQR)	3.8 μg/L (6.9)	1.3 μg/L (2.6)	9.7 μg/L (4.3) *p* = < 0.001

There was a total of 15 TBIs classified as SRC; seven of which were ultimately proved to be GH deficient. Four patients had documented evidence of other pituitary deficits, which were either central hypothyroidism, central hypogonadism, or both. Of these four patients, one of them had sustained a SRC, though this individual was known to have a history of anabolic steroid use.

**Fig. 1:**
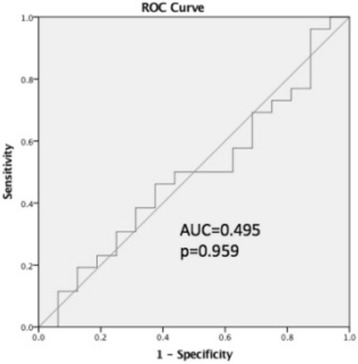
Receiver operating characteristic curve of serum IGF-1 level for diagnosis of growth hormone deficiency by dynamic testing

For each patient, IGF-1 status was correlated with GH status after stimulation testing (Table 2). Patients were labelled as having “low” IGF-1 if the value fell below the assay reference range for matched age and gender, and “normal” IGF-1 if value was within this reference range. Baseline IGF-1 level was not available for four patients in GH deficient group and two patients in the non- GH deficient group. All but one patient had normal IGF-1 levels, and the single patient with low IGF-1 level tested negative for growth hormone deficiency with a peak GH of 50.2 μg/L. Mean IGF-1 z-score was lower for patients in the non-GHD group, but this was non-significant (*p* = 0.979). IGF-1 Z-scores’ ability to diagnose GHD were examined by ROC curve analysis, shown in Fig. 1. ROC analysis showed AUC of 0.495, *p* = 0.959, confirming lack of diagnostic utility.

**Table 2:** IGF-1 and GH status

	GH deficient	Non-GH deficient
IGF-1 low	0	1
IGF-1 normal	25	15
Median z-score	−0.214	− 0.060 *p* = 0.979

## Discussion

Our results demonstrate that baseline serum IGF-1 level had no value in predicting GH deficiency, emphasizing the need for dynamic testing in this population. These findings contradict those of Zgaljardic et al. [7] There are several differences between our respective studies that may account for this. Our study includes an older patient population, and the majority of our subjects (48/62) sustained mild TBI, while the previous paper only included patients with moderate and severe TBI. Because almost all of our patients had normal IGF-1 levels, we cannot exclude the possibility that a truly low IGF-1 level, if present, may have a high specificity for true GHD as has been seen in other hypopituitary states. However, in our data, the single patient with overtly low IGF-1 was not GH deficient and as a group, the non-GHD patients tended to have lower IGF-1 values than the GHD patients and so it may be that in TBI patients, IGF-1 measurements of any kind have very poor sensitivity or specificity for true GHD. Importantly, the IGF-1 assay used at our centre differs from that used by Zgaljardic et al.; significant variability amongst different commercial IGF-1 immunoassays has been highlighted previously [12]. Furthermore, the different reference interval for each assay is a source of further complexity regarding the comparison of results between studies [13]. While reference intervals need to be method-specific, there is considerable variability in how reference ranges are derived for IGF-1 and may lead to different interpretations for the same patient [14]. Given this variability, it may require multicenter studies to validate robust reference ranges for specific IGF-1 methods to allow for better correlation between studies [12, 15]. Another factor can be due to the regulation of IGF-1 and its association with binding proteins, particularly with IGF binding proteins, whereby IGF-1 immunoassays have relied on displacement of IGF-1 from IGF binding proteins for proper measurement. Further advances in IGF-1 measurement by tandem mass spectrometry may be more specific and ensures the displacement of binding proteins by assay design. However, tandem mass spectrometry methods will similarly require proper validation of reference ranges as well as standardization [16]. In addition, the tandem mass spectrometry method may miss certain IGF-1 protein variants which have unknown functionality and will be unable to distinguish from wild type individuals [17]. Nevertheless, a repeat study that employs the use of a well-developed tandem mass spectrometry method for IGF-1 is warranted and will address the role of assay specificity in addition to variability in reference ranges. Taken together, the limitations in IGF-1 assay standardization and the results presented herein suggest the use of more specific dynamic testing of GH reserve.

Our findings add to a growing body of literature documenting pituitary dysfunction secondary to sport-related concussion. This can present with isolated or multiple pituitary hormone deficits, with GHD being the most common isolated deficit [18, 19]. While previous literature has implicated contact sports involving repetitive head trauma [4], our study includes two patients who developed growth hormone deficiency following an isolated concussion sustained while cycling. Therefore, even individuals who sustain a single concussion from non-contact sports appear to be susceptible to developing some degree of pituitary dysfunction.

Our study has several methodological limitations. Our results may not be generalizable to other institutions that use different IGF-1 assays, as we have outlined above. The referral process was not standardized; decisions about which patients are referred for endocrinology assessment reflect local practice patterns which may differ at other centres. For each patient, only a single dynamic test of GH reserve was performed, however, additional testing is not currently feasible due to cost and resource limitations. In the absence of other pituitary hormone deficits or obvious structural abnormalities in the pituitary, expert opinion suggests that two dynamic tests of GH reserve should be performed in order to confirm isolated GHD [20]. However, an FDA or Health Canada-approved GHRH method is not available in North America for GHRH stimulation testing, while insulin hypoglycemic testing may not be widely available at many centres and thus multi-modality testing may not be possible. In the absence of a “gold standard” diagnostic reference, it is also unknown as to whether the combination of dynamic GH stimulation tests truly improves diagnosis. In theory, diagnostic specificity may be improved but at an unknown loss of diagnostic sensitivity; this requires further study. We are unable to obtain BMI data from our EMR, which may have diagnostic implications as BMI can influence peak GH response, though the evidence on this matter is conflicting [7].

## Conclusion

Our study has demonstrated the need for dynamic testing of GH reserve for patients with TBI who are suspected to have GHD at our centre. Individual measurement of IGF-1 levels and interpretation of the results according to method-specific reference ranges is ineffective to screen for GH deficiency in patients with TBI. Sport-related concussion, not just repetitive SRC of any nature appears to be a risk factor for subsequent GHD.

## Abbreviations

GH: Growth hormone
GHD: Growth hormone deficiency
GST: glucagon stimulation test
hGH: human growth hormone
ITT: insulin tolerance test
SRC: sport-related concussion
TBI: traumatic brain injury
